# The Urine of the Horse in Pneumonia

**Published:** 1901-02

**Authors:** M. D. Hoge

**Affiliations:** Richmond, Va., Professor of Pathology, University College of Medicine.


### THE URINE OF THE HORSE IN PNEUMONIA.

By M. D. HOGE, Jr., M.D., Richmond, Va.,

Professor of Pathology, University College of Medicine.

Believing that if we all make some contribution to veterinary science, however small it may be, still when the aggregate is taken the result may possibly be of considerable value.

I have selected for my subject the examination of the urine of a horse suffering from a typical case of pneumonia. Through the kindness of Dr. A. F. Smith, of this city, I was enabled to secure a sample from an animal—a well-made, heavy-built horse, 15¾ hands high, weighing about 1200 pounds. It was obtained on the seventh day of the disease, the temperature registering 102½ F. that morning.

The color of the urine was a light amber and quite transparent; the specific gravity 1.012, which was considerably below the normal, that is, 1.036. The total quantity in the 24 hours was somewhat increased, due probably to the fact that the patient had been taking 30-drop doses of tincture of digitalis at frequent intervals.

The reaction was *acid*, partly due to the lack of the salts of those food substances, which being easily converted into carbonate of potash, produce the normal alkaline reaction in herbivord, and partly to the fact that animal food (such as eggs) and stimulants were given to keep up the strength. While the acidity was distinct and pronounced, it was not strong—being equal to plus 8 of a decinormal sodic hydrate solution.

Sugar, by Haines' copper solution and Boettger's bismuth test, was negative.

Albumin was found to be present in amount to 2 per cent. by bulk.

Urea, as estimated by the amount of nitrogen set free by the hypobromite of soda, was 4 grains per ounce.

Phosphates to the extent of 4-10 of 1 per cent. were found; indican only a trace.

Chloride of sodium by the nitric acid and silver nitrate solution was found to be almost entirely absent. This is an interesting coincidence, because the same thing is found in man in pneumonia; in fact we are accustomed to say, that in this disease when the chlorides are absent on the approach of the crisis a fatal prognosis is justified.

When we know that the average daily amount of salt excreted by the horse is 85 grains, then its reduction to a mere trace, taken in connection with an extensive fibrinous exudation must be regarded as of pathonomonic importance.

Microscopically, there were evidences of parenchymatous nephritis as shown by the presence of fatty degenerated epitheleum and the fine granular casts.

No uric acid, oxalate of lime crystals or blood cells were found.

No one feels more than I, the incompleteness and crudeness of this short paper, and my excuse is the brief notice given me for its preparation, and the difficulty in obtaining the necessary material. However, I deem the subject worthy of the investigation of the members of our Society, if it leads us to a more careful and possibly useful means of forming a diagnosis and prognosis in this disease.

308 *East Grace Street.*

